# The perspectives on advance care planning of older people with psychotic illnesses and their carers

**DOI:** 10.1007/s41999-025-01161-8

**Published:** 2025-03-14

**Authors:** Anne Wand, Aspasia Karageorge, Yucheng Zeng, Roisin Browne, Meg Sands, Daniella Kanareck, Vasi Naganathan, Anne Meller, Carolyn Smith, Carmelle Peisah

**Affiliations:** 1 Specialty of Psychiatry, Faculty of Medicine and Health, University of Sydney, Sydney, Australia; 2 School of Psychiatry and Mental Health, Faculty of Medicine and Health, University of New South Wales, Sydney, Australia; 3 Brain and Mind Centre, University of Sydney, Sydney, Australia; 4 Faculty of Medicine and Health, University of Sydney, Sydney, Australia; 5 Macquarie University, Sydney, NSW Australia; 6 Capacity Australia, Sydney, Australia; 7 Faculty of Medicine and Health, Prince of Wales Clinical School, University of New South Wales, Sydney, Australia; 8 Prince of Wales Hospital and Community Health Service, South Eastern Sydney Local Health District, Sydney, NSW Australia; 9 Older Peoples Mental Health Service, Prince of Wales Hospital, Randwick, NSW Australia; 10 Centre for Education and Research on Ageing, Concord Repatriation Hospital, Concord, NSW Australia; 11 Older Peoples Mental Health Service, KGV Level 7, Missenden Rd, Camperdown, NSW 2050 Australia; 12 Clinical Nurse Consultant, Advance Care Planning (retired), Prince of Wales Hospital and Community Health Service, Sydney, Australia; 13 Carer Advocate for Advance Care Planning, Sydney, Australia

**Keywords:** Palliative care, Healthcare systems, Psychosis, Qualitative, Death and dying

## Abstract

**Purpose:**

To examine the attitudes, experiences, and perceived facilitators and barriers to Advance Care Planning (ACP), of older people with schizophrenia and other psychotic illness and their carers.

**Methods:**

Older people with a psychotic illness and carers were recruited from public mental health services in Sydney, Australia. Semi-structured interviews to explore attitudes, experiences, and perceived barriers and facilitators to ACP were conducted by an external clinician. Consumers’ medical, psychiatric and drug health diagnoses were recorded and they completed cognitive and symptom rating scales. Reflexive thematic analysis was used to analyse the transcripts of interviews, within an interpretive description framework.

**Results:**

Thematic saturation was achieved with 12 consumers and 5 carers. Emergent themes from consumer interviews were (i) ‘What is ACP?’; (ii) ‘I have not done ACP because…..’; (iii) ‘I want to do ACP’; (iv) ‘If I was to do ACP I would need..’; and (v) ‘Mental health clinicians have the skills to help me with ACP’. Carer themes included (i) ‘We do not participate in ACP’, (ii) ‘I want to participate in ACP’, and (iii) ‘Key clinician skills are needed’. There was convergence of themes from both groups.

**Conclusion:**

This study demonstrates that older people with a psychotic illness can express views regarding ACP, despite ongoing symptoms of psychosis, cognitive impairment and mild–moderate severity of illness. The emergent themes highlight opportunities to intervene to overcome barriers to ACP, including education for both participant groups and clinicians, practical considerations and the need for all to collaborate, including with primary care.

## Key summary points

**Aim:**

The aim of the study was to qualitatively examine the attitudes, experiences, and perceived facilitators and barriers to Advance Care Planning (ACP), of older people with psychotic illness and their carers.

**Findings:**

Older people with psychotic illnesses do not know about ACP but are interested, want to be involved, and identified how this could be facilitated by clinicians they trust. Their carers raised the complexity of ACP in light of cognitive impairment, the culture and personal histories of consumers and reality of busy health professionals; identifying carer roles supporting consumers and assisting clinicians with cultural brokerage.

**Message:**

Older people with psychotic illnesses are able to express views regarding ACP and, alongside their carers, highlighted ways of overcoming barriers to ACP, including education, practical considerations and collaboration with primary care.

## Introduction

Advance Care Planning (ACP) is a process whereby a person iteratively discusses and expresses preferences for future treatment aligned with their wishes, values and life goals [1]. Alongside promoting autonomy, ACP is associated with other beneficial outcomes including less aggressive medical care and earlier hospice referral for patients, and better quality of life and emotional wellbeing for caregivers [2–5]. Quality care towards the end of life is a human right [6, 7]. However, this right may not be realised in people with serious mental illnesses such as schizophrenia, despite often disproportionate need conferred by medical morbidity and premature mortality [8, 9]. Moreover, people with serious mental illnesses are more likely to experience stigma and fragmented care [10, 11] and have worse symptom management, including of pain [12, 13], towards the end of life and are less likely to access specialist palliative care [13, 14].

People with serious mental illness, especially those who are older, often have limited opportunities to engage in ACP discussions [15–18]. Previous studies have either indirectly explored perspectives of people with mental illness on ACP through hypothetical vignettes presented within questionnaires [19, 20], or examined practical aspects of palliative care implementation in people with incurable medical conditions and mental illness [21, 22]. There has been relative neglect of the issue of ACP more generally with people with psychotic illnesses such as schizophrenia [18] with various reasons postulated [23–25]. This is particularly true amongst older adult populations with psychotic illnesses, and those with persistent or severe symptomatology, which is important because this is often perceived as a barrier to ACP. Specifically, the symptom burden and severity of mental illness of study participants has either not been reported [20, 21, 26], or participants were recruited during illness recovery [22]. This has limited interpretation of study findings to real world clinical populations.

Furthermore, with exception of one small study with a palliative care focus which include three carers [21], the perspectives of carers of older people with a psychotic illness are largely absent. As such, the voices of older adults with serious mental illness such as schizophrenia and their carers remain largely unheard.

The aim of this study was to qualitatively explore the attitudes, experiences, and perceived facilitators and barriers to ACP, of older people with schizophrenia/other psychotic illnesses and their carers.

## Materials and methods

### Study design

This qualitative study used semi-structured individual interviews with older adults with schizophrenia or other psychotic illness (consumers), and—separately—carers. A semi-structured interview was used flexibly by the facilitator to explore their attitudes, experiences, and perceived barriers and facilitators to ACP.

### Population and participants

Participants were recruited from public mental health services across three local health districts in Sydney, Australia. Three participant groups were included; consumers, their nominated carer, and mental health clinicians. Qualitative data from mental health clinicians is reported elsewhere [27]; this paper reports data from consumers and carers. Consumers included persons aged ≥ 55 with a diagnosis of schizophrenia or another psychotic illness and able to provide informed consent (or for those lacking capacity, participants included those who assented (i.e. agreed to participate), were able to participate in supported decision-making and their proxy decision maker provided informed consent). For all participants, involvement in the study was considered unlikely to adversely affect their mental health, as determined by a health professional. Carers were adults aged ≥ 18 who have a relative/friend aged ≥ 55 with schizophrenia/other psychotic illness and were able to provide informed consent.

### Setting, participant recruitment and consent process

Eligible participants were referred to the study via their clinicians (e.g., case manager or psychiatrist). All participants with capacity to do so provided informed consent to participate, or for those lacking capacity, their proxy. Consent was given in writing or online e-consent. Individual interviews were conducted via videoconferencing or in person at a community health centre or residential aged care facility by the same facilitator (RB). Recruitment occurred between February and April 2024, until data saturation was reached in each participant group.

### Data collection

For consumer participants, demographic information, medical diagnoses, psychiatric and drug health diagnoses were collected from their Electronic Medical Record (EMR). Immediately before or after the interview, cognitive and symptom rating scales were administered in person by the trained project coordinator (YZ). Symptom rating scores included the Brief Positive and Negative Symptom Scale (PANSS)[28] and Clinical Global Impression Scale (CGI)[29]. Cognition was assessed with the Montreal Cognitive Assessment (MoCA)[30] or Rowland Universal Dementia Assessment Scale (RUDAS)[31], for participants whose first language was not English.

For carer participants, information about gender, relationship to the person with schizophrenia/other psychotic illness and whether they lived with the person were noted. Additional demographic data regarding the participant’s ethnicity and religion were collected retrospectively as an iterative decision because cultural background emerged as an important contextual factor in the thematic analysis.

A semi-structured interview guide was used flexibly by the facilitator to explore the attitudes, experiences, and perceived barriers and facilitators to ACP. Interviews were audio recorded and transcribed. Professional healthcare interpreters were used for non-English speaking participants.

### Data analysis

Reflexive thematic analysis of interview transcripts was conducted [32–35], grounded within an interpretive description framework. Instead of producing primarily theoretical insights, this qualitative approach aims to describe phenomena in a way that applies to clinical practice to inform and improve service delivery. This involves utilising informed questioning of participants to explore explanations and meanings, and subsequent inductive analysis, all aimed at producing practically useful “descriptions” which inform clinical understanding [36, 37]. Developed specifically for health contexts, this practice-oriented method provided an appropriate scaffolding for the thematic analysis to support the needs-based implementation of ACP.

Data were imported into NVivo 12 for analysis. An inductive approach was used to code themes, recognising that meaning emerges from the interaction of coder and text. Initially, analytic categories and primarily semantic themes were generated to remain close to the participants' voices and experiences. Using the interpretive description model, categories were developed to address each research question. Semantic codes were used to group similar experiences described in the transcript data. The primary coder (AK) performed the initial coding, while higher-order coding was conducted by lead investigator (AW) and senior author (CP). To enhance the reciprocity and transparency of the results, participants were invited to review a summary of the thematic analysis, contributing to further refinement of the analysis [38].

The primary coder reviewed and considered hierarchical relationships between the codes as transcripts were analysed and predominantly semantic codes created. These groupings were refined through continuous comparison between codes and data as transcripts were read and re-read, leading to the generation of themes and sub-themes. Subsequently, more latent themes—ideas and concepts underlying the interviewees' words—that captured the nature of the data and directly addressed the research questions emerged.

### Reflexivity

The researchers recognise their influence on data analysis, which is partly shaped by their personal and professional backgrounds [33, 35]. The primary coder, AK, is a clinical psychologist and qualitative researcher. The interviewer, RB, is a senior occupational therapist specialising in older adults and provisional psychologist. The project coordinator, YZ, is a final-year Master of Clinical Neuropsychology student. Senior author CP is a human rights and old age psychiatrist and long-time advocate for ACP. The lead investigator, AW, is an old age psychiatrist working in a publicly funded, community-based Older Persons Mental Health team. The research team also includes a specialist in palliative medicine with experience integrating ACP into electronic medical records (MS), a retired ACP nurse consultant (AM), a carer advocate for ACP with older adults (CS), an academic geriatrician with research and clinical experience in ACP (VN), and a team leader of an older persons mental health service and senior social worker (DK). The most subjective influence on coding was the team's strong advocacy for ACP.

### Ethical considerations

The study was approved by the Human Research Ethics Committee—Concord Repatriation General Hospital of the Sydney Local Health District (2023/ETH02283).

## Results

### Participants

Data saturation occurred with 12 consumers (interview duration 8–29 min, mean 18 min) and 5 carers (duration 15–41 min, mean 27 min). Two consumers who lacked capacity to consent assented to being involved using supported decision-making. Their proxy provided informed consent for participation. Demographic characteristics of the participants are summarised in Tables 1 and 2.

**Table 1:** Demographic characteristics of consumers

Characteristic	N (%) n = 12
Age, years	Mean: 69 (SD = 4.8), range: 60–75
Gender	
Male	7 (58%)
Female	5 (42%)
Ethnicity	
Australian	4 (33%)
Aboriginal Australian	2 (17%)
Chinese	1 (8%)
Egyptian	1 (8%)
New Zealander	1 (8%)
Mauritian	1 (8%)
Sri Lankan	1 (8%)
Vietnamese	1 (8%)
Religion	
No religion	5 (42%)
Protestant Christian	4 (33%)
Roman Catholic	2 (17%)
Buddhist	1 (8%)
Marital status	
Never married	7 (58%)
Married	3 (25%)
Divorced	2 (17%)
Primary support type*	
Residential aged care facility	3 (25%)
National Disability Insurance Scheme	3 (25%)
Family	3 (25%)
None	3 (25%)
Home care package	2 (25%)
Accommodation type	
Community housing (public)	5 (42%)
Private housing	4 (33%)
Residential aged care facility	3 (25%)
Psychiatric and drug health diagnoses^<^	
Schizophrenia	6 (50%)
Depression with psychotic features	3 (25%)
Schizoaffective disorder	2 (17%)
Very late onset schizophrenia-like psychosis	2 (17%)
Substance use disorder	1 (8%)
Depression	1 (8%)
Number of other medical diagnoses	Mean: 5.7 (SD = 3.3), range: 0–11
Symptom/cognitive test scores	
Brief PANSS (N = 12)	Mean: 13.8 (SD = 6.4), range: 7–27
MoCA (N = 11)	Mean: 20.8 (SD = 4.5), range: 13–27
RUDAS (N = 1)	17
CGI (N = 12)	Mean: 3.1 (SD = 1.3), range: 0–5

^*^Some consumers had > 1 type of support

^<^Some consumers had more than one diagnosis

PANSS = Positive and Negative Symptom Scale

The Brief PANSS is rated across six symptoms (e.g., delusion, unusual thought content), with each scored from absent (1), minimal (2) through to extreme (7)

MoCA = Montreal Cognitive Assessment

A score ≥ 26/30 is considered normal

RUDAS = Rowland Universal Dementia Assessment Scale

A score ≥ 26/30 is considered normal

CGI = Clinical Global Impression Scale

The CGI severity of illness scale is rated from 1 = normal, not at all ill, 2 = borderline mentally ill, to 7 = among the most extremely ill. A score of 0 = Not assessed

**Table 2:** Demographic characteristics of carers

Characteristics	Mean (%) n = 5
Gender	
Male	3 (60%)
Female	2 (40%)
Ethnicity	
Vietnamese	2 (40%)
Australian/UK	1 (20%)
African	1 (20%)
Egyptian	1 (20%)
Religion	
Protestant Christian	2 (40%)
Roman Catholic	1 (20%)
Buddhist	1 (20%)
Atheist	1 (20%)
Carer relationship to consumer	
Sibling	2 (40%)
Paid carer (including NDIS)	2 (40%)
Child	1 (20%)
Informant co-resident with consumer	
Yes	3 (60%)
No	2 (40%)

NDIS = National Disability Insurance Scheme

### Thematic analysis

#### Consumers

Five themes emerged from analysis of consumer interview transcripts: (i) ‘What is ACP?’; (ii) ‘I have not done ACP because…..’; (iii) ‘I want to do ACP’; (iv) ‘If I was to do ACP I would need..’; and (v) ‘Mental health clinicians have the skills to help me with ACP’.

(i) ‘What is ACP?’

Some consumers had no idea what ACP was, confusing it with euthanasia and other documents such as wills and powers of attorney, assuming it could only be made with lawyers.

“Haven't heard of it before, today's the first time I've been introduced to it. Is it kind of similar to the term euthanasia or is it different?” (female, 67)

“Has it got the legal aspect to it? ….So would it involve a lawyer?” (female, 73)

(ii) ‘I have not done ACP because…’

A range of subthemes emerged which explained the lack of engagement with ACP:

‘It’s not on my radar’

“I haven't thought about it but it would be important wouldn’t it?” (female, 71)

“Nobody ever addressed it ….Today's the first time.” (female, 67)

‘I didn’t know it was my choice’

“I hope I will be able to have a plan and it would be listened to […] but I fear [doctors] do what they think best and not what I think best. Does [an ACP] help or what? They take notice or not?” (female, 71)

‘Underestimation of the consumer’

“They [the medical team] might not think you've got a brain.” (female, 71)

‘My GP is too busy’

“There just hasn't been that opportunity for a one to one with people who can share the advanced planning and talk about it and explain it. [The GP] is not going to address it because there's so much for them to run through when they see you [already].” (female, 67)

A series of discussions was proposed to remedy this issue;

“If I could have a couple of appointments, instead of just one.” (female, 61)

‘It’s not time yet, I’m not dying’

Participants assumed that doctors only did ACP with those at the end of their life.

‘ACP costs money’

“I have no idea what the cost would involve [to see the GP for it].” (female, 67)

(iii) ‘I want to do ACP’

Conversely, several subthemes emerged which reflected identified benefits for the individual and their family:

‘It would be in my best interests’

“The purpose [of an ACP] would be to look after my wellbeing.” (male, 75)

“It will help me a lot.” (male, 69)

“[I] know what I want, and what I don’t want.” (female, 61)

“Because [an ACP] gets things clear. It gets things less mystified.” (male, 68)

‘Peace of mind for having my wishes respected’

“If you don't have these conversations, the wishes of the patient can’t be fulfilled …. and the carers and the family need to know exactly where they’re coming from, what they want done.” (female, 67)

“[ACP ensures that] they respect my wishes and do not do extra treatment and things.” (female, 73)

“I don't really know what will happen to me as I get older.….. I've got nobody to care for me …..so it's always good to know what lies in the future.” (male, 68)

“It's good that you're open about it before you come to that [end-of-life] situation. That you have addressed it, put it all down in writing.” (female, 67)

‘Relieve family of burden’

“If anything happens to me .… everything's organised for her [my daughter]… so she knows what to do …. she’d know what my wishes were before anything happened.” (female, 60)

“Whether you are in a situation where you mentally can't make decisions for yourself and where you have to give your family, your husband or your next in kin to make the decisions for you….. it's good that you're open about it [ACP], before you come to that situation you have addressed it, put it all down in writing.” (female, 67)

(iv) ‘If I was to do ACP I would need….’

Subthemes emerged regarding consumers preferences for ACP and practical aspects of implementation:

‘It requires trust that it will be implemented’

“I think that's just like a personal thing, it's between your family and your carers or whoever is actually executing it for you. So you have that trust with your family and your medical team and you know it will be done, you know it will be executed….it's something based on trust.” (female, 67)

‘I would want everyone involved’

“I think family. Local GP …..the [mental health clinician].” (female, 61)

“....their doctor, their social worker, their ACAT team.” (male, 69)

“And an institution, if I go to one. Like a nursing home.” (female, 73)

“The GP. The doctors. The psychiatrist.” (male, 68)

“It would involve the doctor and the next of kin or family member.” (female, 73)

‘Speaking to a professional about ACP’

“…because I've got nobody else to discuss this with. There are days where I don't speak to anyone …. So [it would be] good to talk to someone about advance care planning.…sometimes you need to have a conversation with a health professional.” (male, 68)

‘Choosing where I do ACP’

“At my house ….. with my wife and children.” (male, 68)

“At [deidentified place]… because that’s where my daughter lives.” (female, 60)

“It could be on a visit to the doctor… [Family] could come in and we could all talk about it together.” (female, 61)

‘Do it early’

“It's good that you're open about it before you come to that [end-of-life] situation. That you have addressed it, put it all down in writing.” (female, 67)

"Mental health clinicians have the skills to help me with ACP"

“Skills in mental health, with going through schizophrenia. No.. [other skills in particular].” (female, 60)

“The professors, doctors, social workers .... you wouldn't [need special skills] in your positions, you’d have that skill already.” (male, 69)

Generic supportive skills were also important;

“Insight, caring, sympathy.” (male, 68)

#### Carers

Themes which emerged from carer interviews included (i) ‘We do not participate in ACP’, (ii) ‘I want to participate in ACP’, and (iii) ‘Key clinician skills are needed’.

(i) ‘We do not participate in ACP’

Interviews with carers identified several subthemes regarding why they do not participate in ACP with the consumer:

‘Death and dying are too complex and confronting’

“At the moment we are not really wanting to address it because it's too disturbing for us… and sometimes we don't see eye to eye so I just don't talk about … I just keep quiet just to keep the peace.” (sister)

“In the role that I work there is a department that does those things, but it's quite a delicate matter which needs timing and a proper conversation to make sure it's not misunderstood. So short answer no [I don’t discuss ACP].” (female, paid carer)

‘It might upset them’

Fears of causing harm through ACP were raised;

“…..we just want to keep it calm for her and give her some hope. But talking about advance care planning, the eventuality is like you haven't got much time to live and it's too disturbing and she's already thinking that, so we don't want to add stress to how she's been feeling.” (sister)

‘Nobody brings it up’

Carers expected clinicians would have raised ACP;

“Whenever I see specialists, or even the doctor, or GP, or last year my father had to go to the hospital, but I have never heard them talk about advance care planning.” (son)

“Nobody [GP/other doctors] has ever mentioned it, ……no one has sat down with me and communicated it.” (male, paid carer)

‘General practitioners (GPs) are too busy and unskilled’

“GP is just 5, 10, 15, 20 minutes and deal with the medication, they haven't got the patience for it or the experience to talk about advance care planning….. Their role is just make referral and follow instructions about specialists telling them what to do…” (sister)

‘ACP is only needed for people who are dying’

“It's difficult to imagine the seriousness of a condition… But I guess …. once he understands it’s a serious health condition, [then I might discuss ACP].” (brother)

“At the moment she has got better….. But I think when she gets unwell like she was before then….. we have no choice [but] to do ACP….we just want to give her a little bit more peace of mind that she has got better, so we don't want to confront that aspect until we have to.” (sister)

There was a divergent, conflicting theme that it was too late to engage in ACP when the person is dying;

“It's too complex right now and doesn't really make any difference. You know, she’s probably gonna die soon.” (male, paid carer)

‘Not appropriate if cognitively impaired’

“When there's impactful cognitive decline, it feels almost unkind to try and throw them into [an ACP] conversation.” (male, paid carer)

“[It would be] mentally straining to go through that process [with the consumer] so I’m like 100% sure she won’t remember the next day.…. when there's any sort of …..impactful cognitive decline… that becomes a very, very difficult grey area, in terms of the initial conversation. That's generally for the relationship between a GP and a patient.” (male, paid carer)

(ii) ‘I want to participate in ACP’

Several subthemes emerged regarding why carers would like to participate in ACPs with consumers:

‘Families have important roles in ACP’

“He's open minded [but] how adjusted he is about that conversation will depend on how strongly he feels support and our presence.” (brother)

“My father, when he sees the Vietnamese GP sometimes he really wants to talk to the GP. But he is scared, or he is shy, or he doesn't talk. So he normally talks with me at home first, and then when I bring him to GP or doctor, I will talk to the doctor.” (son)

“Being from another community, not the mainstream community, family is quite important and surrounded by family and loved ones is quite important.” (sister)

Whereas professional carers considered ACP outside of their roles.

"I don't think it's the right thing for me to do because there is that therapeutic relationship…. It's something maybe that we can assist to link or to research who's available to help in doing that.” (female, paid carer).

‘It will keep the consumer and me informed’

“I think it will be very useful… I think it's important to keep them informed. I'd like to do that.” (male, paid carer)

“Yeah, I'm happy with [talking to consumer about ACP]. It will help me understand more.” (son)

‘Empowerment and giving voice’

“It's quite hard to have those conversations when someone is unwell and sometimes decisions have to be made, and [ACP] is sort of like giving the person a voice, a choice, while they're still able to.” (female, paid carer)

‘Peace of mind in having wishes respected’

“These conversations obviously are quite important. And once planning is in place, or once decisions are made…. I think people feel lighter and more liberated, and sort of confident in life a bit more, that their own wishes are followed or applied, implemented.” (brother)

(iii) ‘Key clinician skills are needed’

Carers recognised the skills needed in clinicians undertaking ACP with consumers:

‘Patience and attitude’

Carers noted particular qualities in mental health professionals which support ACP.

“The attitude is really important, the enthusiasm. [The mental health clinician] is so close, friendly, and spends time with [the consumer]. They care. He doesn't mind to talk to my father [the consumer]. He’s kind. We really appreciate [that he takes time]. It fluctuates quite a lot. Some days you can have a decent conversation, and some days he cannot understand.” (son)

‘Cultural understanding and language’

Carers may assist as cultural brokers;

“Given that we're coming from a different cultural background, I think the barrier is a lack of understanding the cultural aspect of [dying and decision-making]. Also the language aspect as well, and are [the clinicians] culturally sensitive enough to understand the cultural information that [the consumer] has given, that might not fit within the norm or expectation?” (sister)

“My mother, she doesn't understand English at all [therefore I need to be involved].” (son)

‘Understanding trauma histories’

“People from refugee background there's a whole heap of trauma that they have had that is just hidden….. If they [clinicians] don't pick the right time [for ACP] it's retraumatising the individual.…. we don't want to retraumatise her. Not only that but retraumatise the family as well. So there's a lot hidden or untreated trauma that the refugee community or people who've been through war and stuff been through. So if it’s not done sensitively it actually retraumatise them without even knowing.” (sister)

'Flexible opportunities to do ACP'

“I prefer if we can do online because if we have to go to [hospital] or somewhere… the time will be a little hard, it’s not flexible.” (son)

### Commonalities and convergence

Convergent themes emerging from both groups included previous lack of engagement with ACP because no-one had raised it, beliefs that ACP was only relevant to the dying, and busy GPs. Simultaneously, there was convergence in a desire to engage in ACP, with acknowledgment of benefits for respecting consumer preferences and wishes, providing peace of mind (for consumers and carers), and relieving family burden. Both groups noted that ACP needs flexibility with practical aspects (e.g. location), time, and skills, and identified mental health clinicians as having these skills. Carers recognised their important role in ACP given their understanding of the consumer’s cultural and personal (for some, trauma) background. They also expressed fear of causing harm if ACP was not done sensitively and at the right time. Concerns about ACP with cognitively impaired consumers were also raised.

## Discussion

This qualitative study is novel in examining and triangulating the experiences and perspectives of older consumers with psychotic illnesses and their carers on ACP. Themes emerging from consumer interviews were (i) ‘what is ACP?’; (ii) ‘I have not done ACP because…..’; (iii) ‘I want to do ACP’; (iv) ‘If I was to do ACP I would need..’; and (v) ‘Mental health clinicians have the skills to help me with ACP’. For carers, the core themes were (i) ‘We do not participate in ACP’, (ii) ‘I want to participate in ACP’, and (iii) ‘Key clinician skills are needed’. There was convergence between many of these themes, and those identified in the carer group. Our study uniquely situates the consumer’s viewpoints within their cognitive and psychiatric symptom context and is inclusive of nursing home residents and people from diverse cultural backgrounds, often excluded from research but identified as critical to examine [21]. Importantly, despite evidence of cognitive impairment (mean MoCA/RUDAS scores 21/30 and 17/30, respectively) and ongoing psychotic symptoms (ranging from mildly to markedly ill), consumers were well able to articulate their views on ACP [39]. This directly challenges previously reported misconceptions that people with serious mental illness or cognitive impairment cannot meaningfully participate in discussions about ACP [39] and addresses the call for research involving patient and carer views [10, 40].

Although lack of familiarity with ACP was striking among consumer participants in this study, studies of community-dwelling older adults and residents of aged care facilities in Australia have reported similar low awareness of ACP [41]. Even amongst middle-aged people with mental illness who had considered their end-of-life preferences, few had discussed them with their doctor, and even fewer had documented them [19]. Consumers assumed that if ACP was relevant and important, healthcare professionals would have raised it with them. This represents a missed opportunity to promote equal access to decision-making and person-centred care at the end of life, a universal human right [7]. The absence of such discussions also fed misconceptions that ACP was only for the dying. This highlights the important role healthcare professionals have in awareness-raising and supporting ACP with people with mental illness [10], particularly within mental health services, who are perceived by consumers and carers in this study as caring, and having the skills and time to do so.

There were numerous advantages to ACP highlighted by consumers, including the promotion of best interests, piece of mind knowing their wishes will be respected and relieving family of this decision-making burden. Interestingly, this appears to be the first empirical study directly exploring the benefits of ACP as perceived by people with mental illness themselves, with other work largely focused on the complexity and barriers to quality end-of-life care in people with serious mental illness [10, 21, 39, 42]. Our findings align with calls for the promotion of self-determination, autonomy, dignity and respect for will and preferences of older people with mental illness, especially in the face of global ageism and mentalism (discrimination of people with mental illness) [7, 43].

Although consumers wanted to engage in ACP, they identified several barriers. Many such barriers related to lack of knowledge and awareness about ACP, including misconceptions that it must cost money or require a lawyer. As stated above, some consumers assumed ACP was only for those imminently dying, perhaps reflective of current practice [14, 44, 45], although identified as needing to change [7, 20] Issues of stigma and perceptions of clinicians underestimating consumers’ ability to engage in ACP were reported here, similarly identified in studies of people with both severe mental illness and an incurable physical condition [21] and clinicians [17, 46]. Practical barriers such as lack of time to discuss ACP in primary care were also noted here and in the literature [47], and have been echoed by mental health clinicians as a barrier to their own engagement in ACP with consumers [27].

Consistent with consumer participants, while carers had not been involved in ACP with consumers, they expressed preference for such and recognised the importance of their potential roles. Carers valued being informed and emphasised the importance of giving voice to consumers, both empowering and providing peace of mind to consumers and carers alike. Carer roles of advocacy and expertise in the consumer’s condition, treatment and care needs have been previously identified as important by carers of people with serious mental illness and incurable conditions [21]. In our study, carers emphasised the value of their broad knowledge of language and culture as well as the consumer’s personal background, including trauma histories, to support consumers and facilitate clinicians engaging in nuanced and culturally safe ACP with consumers. This novel finding highlights the importance of engaging culturally diverse participants in research, hitherto identified as a gap in this literature [21].

Common to consumer findings, the various factors precluding carers’ involvement in ACP included presumptions that clinicians would have raised ACP if it was relevant and that it was only relevant to those who are terminally ill. They too identified that GPs may lack time or necessary skills to undertake ACP with consumers. Carers identified the complexity of ACP with people with mental illness and/or cognitive impairment and fear of causing harm, concerns also shared by clinicians [10, 46, 27]. The issue of communication was mentioned in relation to ACP not being raised with consumers and carers. However, in another related study focusing on end-of-life care for people with serious mental illness more generally and an incurable medical condition, carers self-identified identified as ‘ignored experts’ [21]. They perceived their expertise as a carer was largely ignored by clinicians and felt excluded from planning and decision making [21].

### Strategies to facilitate ACP in older people with psychotic illnesses

Themes pertaining to consumer- and carer-identified reasons for not engaging in ACP as well as needs were used to propose strategies to facilitate ACP (Table 3). Key among these were practical considerations such as having sufficient time, and flexibility in the setting, timing and form of ACP. Clinicians should be holistically informed about the older person with mental illness, by leveraging the expertise of carers [21] and through person-centred care [44]. Having the right skills and attitudes, especially in relation to mental illness, were also emphasised.

**Table 3:** Strategies to facilitate ACP

Identified need	Components to be addressed
Educating consumers and carers- Myths to be dispelled	It’s your choice It’s not euthanasia It does not have to be done with a lawyer It can be done without cost When to do ACP- not only for the dying, engage in ACP early [20] N.B This could be delivered with ACP information brochures specifically developed with people with mental illness and their carers and through clinician discussions
Educating clinicians	Assuming capacity to communicate wishes and capacity assessment when needed, rather than under-estimating consumers [see also 27] How to navigate ACP with people with cognitive impairment Recognise that carers may want to be involved (with consumer permission)
Professional skills	Expertise in mental health Strong therapeutic alliance- trust, patience, sensitivity
Supported decision making	Ask consumers about who they would like to support them and involve nominated relatives/friends
Considering culture	Interpreters may be needed to communicate in the language best understood by the consumer Get it right- Families may facilitate understanding of the consumer’s cultural and personal history, including trauma, to engage in ACP sensitively, alleviate anxiety and promote supported decision making
Flexibility	Timing (when to do it) Location of ACP (e.g.at home, in the GP surgery, in appointments with mental health clinicians) Mode of discussion- online or face to face according to consumer preferences
Time	Make a series of shorter appointments Discuss ACP iteratively over a number of occasions
Sharing ACP	With the consumer themselves, family, GP, MH professionals

Consumer voices highlighted the importance of not underestimating older people with a psychotic illness. Despite diverse cultural backgrounds, symptoms of mental illness and cognitive impairment, consumers here demonstrated an ability to discuss ACP and willingness to make such plans. Other studies have similarly shown that people with mental illness, including those with cognitive impairment [20], can express preferences regarding aspects of ACP both through hypothetical vignettes in a questionnaire [19] and direct interviews regarding palliative care [22]. The lived experience of stigma described by consumer participants is reflected by the misconceptions of clinicians, reported previously [17, 46, 27] and carer participants. This must be addressed to ensure that clinicians and carers discuss ACP with consumers and document and share their wishes and preferences. Education is one avenue of promoting awareness and dispelling myths regarding capacity and fears of causing harm.

General practitioners are well-positioned to discuss ACP early with their patients given their longstanding relationships and position of trust, and as they see patients over times when their health is relatively stable [48, 49]. The Position Statement of the Royal Australian College of General Practitioners (2012) states that ACP should be part of routine general practice and has clinical resources to support this on their website (https://www.racgp.org.au/). However, there are identified barriers to implementation in general [48, 49], let alone ACP with people with psychotic illnesses, which is more complex, and requires even more time, as identified by the participants. Regardless of the setting for ACP, primary care, community mental health or hospital-based, the practise of the 5-minute unilateral monologue has been broadly criticised [50]. We support the suggestion of one of the participants to undertake this over several sessions. This approach facilitates implementation for busy clinicians and allows for the iterative discussion intended in ACP [1].

In the emergent themes regarding clinician skills, consumers and carers identified that the expertise of mental health clinicians specifically was directly relevant to ACP with people with psychotic illnesses. They also highlighted broader therapeutic skills such as empathy, patience, and sensitivity and perceived that mental health clinicians had time (or perhaps gave consumers time) to properly discuss ACP. The therapeutic relationship between the mental health clinician and consumer was similarly noted as facilitating ACP in our previous qualitative study with clinicians; however, clinicians did not consider they had time to explore ACP and some did not consider it their role [27].

### Strengths and limitations

Most consumers had capacity to consent to participation in the study (10/12). However, consumers lacking capacity may be among those most likely to be excluded from ACP in clinical practice due to aforementioned assumptions and misconceptions of clinicians [17, 46, 27]. The inclusion of those lacking capacity using supported decision-making was deliberate to ensure the sample was representative and to give these consumers a voice, although they were in the minority. Notwithstanding the importance of canvassing the perspectives of carers, hitherto largely absent from the literature, it is important to acknowledge that this was a privileged sample of interested, caring, involved carers who chose to participate. Thus, they may not be representative of all carers/relatives of people with a psychotic illness, particularly those who may exert undue influence on end-of-life decisions as a form of abuse, or as consequence of carer burnout, noted in other contexts [51]. Further, many older people with mental illness do not have concerned family/friends and rely on healthcare professionals for support regarding end-of-life care [22].

Although this is the first study to objectively measure current psychiatric symptoms and illness severity in order to contextualise consumer responses, the Brief PANSS has not been psychometrically tested [52]. However, the Brief PANSS correlates well with the full PANSS [28] and the CGI scores provided further information regarding the severity of illness in our sample.

Reciprocity and transparency were further strengths of the study enhanced by seeking participant feedback on the initial thematic analysis [38]. This study involved older people with a psychotic illness and their carers, however, the results may be more generally applicable to older people with other mental illnesses [44].

## Conclusions

Older people with psychotic illnesses and their carers have limited awareness of and access to ACP, although they consider ACP important and want to be involved. Their diverse voices highlight opportunities to address the identified current barriers to ACP. Although the focus of ACP has previously been primary care, consumers and carers identify that mental health clinicians may be the preferred healthcare professional to lead these discussions given their specialist knowledge of mental illness and therapeutic skills. Collaboration between primary and specialist care, combined with carer knowledge and direct discussion with consumers—shown here as very able to discuss ACP despite cognitive impairment and symptoms of mental illness—takes time but is likely to be the optimal approach to meeting the need for ACP in this population.

## Data Availability

The raw data supporting the results of this study are not publicly available due to conditions of the ethics approval granted by the Human Research Ethics Committee- Concord Repatriation General Hospital of the Sydney Local Health District.
